# Upregulation of kinesin family member 4A enhanced cell proliferation via activation of Akt signaling and predicted a poor prognosis in hepatocellular carcinoma

**DOI:** 10.1038/s41419-017-0114-4

**Published:** 2018-02-02

**Authors:** Yanlin Huang, Hongbo Wang, Yifan Lian, Xiaojuan Wu, Liang Zhou, Jialiang Wang, Meihai Deng, Yuehua Huang

**Affiliations:** 1 Department of Infectious Diseases, The Third Affiliated Hospital of Sun Yat-sen University, Guangzhou, China; 2 Guangdong Province Key Laboratory of Liver Disease Research, The Third Affiliated Hospital of Sun Yat-sen University, Guangzhou, China; 3 Department of Hepatobiliary Surgery, The Third Affiliated Hospital of Sun Yat-sen University, Guangzhou, China

## Abstract

Hepatocellular carcinoma (HCC) is the third most frequent cause of cancer-related death worldwide, and the molecular pathogenesis and development of HCC are largely unknown. In the present study, we found that KIF4A expression was upregulated in HCC (678 samples, *P* = 2.03E-8) based on a meta-analysis of Oncomine database. We further confirmed that both KIF4A mRNA and protein expressions were overexpressed in human HCC tumour tissues as well as cancer cell lines. Higher KIF4A expression was correlated with poorer overall survival (*P* < 0.0001) and disease-free survival (*P* < 0.0337) in HCC patients. We constructed in vitro KIF4A overexpression and depletion HCC cell models. KIF4A overexpression significantly enhanced cellular proliferation and clonogenic abilities, whereas KIF4A depletion caused a dramatic increase of cells with abnormal chromosome segregation and subsequently resulted in augmentation of apoptosis in HCC cells. In addition, we demonstrated that KIF4A depletion was related to inhibition of Akt kinase activity and induction of intrinsic apoptosis signaling pathway. Taken together, KIF4A may act as a prognostic biomarker and potential therapeutic target in human HCC.

## Introduction

Hepatocellular carcinoma (HCC) is the most common primary liver malignancy and the third most frequent cause of cancer-related death worldwide^1^. The risk factors for HCC oncogenesis include chronic hepatitis B virus (HBV) or hepatitis C virus (HCV) infection, aflatoxin exposure, alcohol or drugs abuse, and metabolic disorders in liver. Despite great advances having been made to increase significantly the detection rate in early stages of HCC patients, the 5-year overall survival rate of patients with liver cancer, particularly in intermediate stage and advanced stage, is still extremely low^2^. On the other hand, although a growing body of related research was performed in order to explain the oncogenesis process of HCC, the molecular basis still remains obscure. Therefore, revealing the complicated molecular mechanism of pathogenesis and development of liver cancer is currently an urgent global health issue.

Over the past decade, there has been an improvement in the classification of the molecular pathogenesis of HCC^3^. Main drivers responsible for tumour initiation and progression have been discovered through genomic analyses. Common mutations affect telomere maintenance (telomere reverse transcriptase (TERT)), WNT pathway activation (β-catenin (CTNNB1)), inactivation of cellular tumour antigen p53 (TP53), etc. Certain genetic and epigenetic events lead to activation of specific signalling pathways, including regulation of cell cycle progression^4^. Large amounts of published studies show that suppressing cell cycle arrest and/or promoting cell cycle transition could boost the proliferation of pre-cancerous cells or cancer cells and are related to HCC tumorigenesis and progression^5,6^. The spindle assembly checkpoint (SAC) is a quality control mechanism that ensures accurate chromosome segregation during mitosis, and is thus important for cell cycle regulation. The SAC delays cell cycle progression until all chromosomes have successfully made spindle–microtubule attachments. Defects in the SAC generate aneuploidy and may facilitate tumorigenesis^7^.

Kinesin superfamily proteins (KIFs) share a conserved motor domain, and there are a class of microtubule-dependent molecular motor proteins that have been shown to participate in multiple cellular activities, including mitosis, organelles and vesicles transportation. In mitosis, the active movement of kinesins guarantees the precise orchestration of mitotic events and normal activity of SAC^8^. Today, 45 human and murine kinesin proteins have been found and classified into 14 families^9,10^. KIF4A, an N-type kinesin belonging to the kinesin-4 family, contains the ATPase/motor domain that binds to microtubules to provide mechanochemical force and plays a pivotal role in chromosome segregation and mitotic spindle organization during mitosis^11^. KIF4A has been found overexpressed in several malignancies, including oral and pulmonary carcinomas, cervical cancer, and breast cancer, and its overexpression is associated with poor prognosis in these cancers^12–16^.

In the present study, we showed that KIF4A is overexpressed in HCC tissues and cell lines, and high level of KIF4A predicts a poor prognosis in HCC patients. Using KIF4A depletion and overexpression HCC cell models, we demonstrated that KIF4A promotes cell proliferation and clonogenic potential, mainly through maintenance of mitotic progression and protection from apoptosis. We proposed that KIF4A could be a diagnostic biomarker and promising molecular target for anticancer therapy in HCC.

## Results

### KIF4A expression increases in HCC tissues and cell lines

**Fig. 1:**
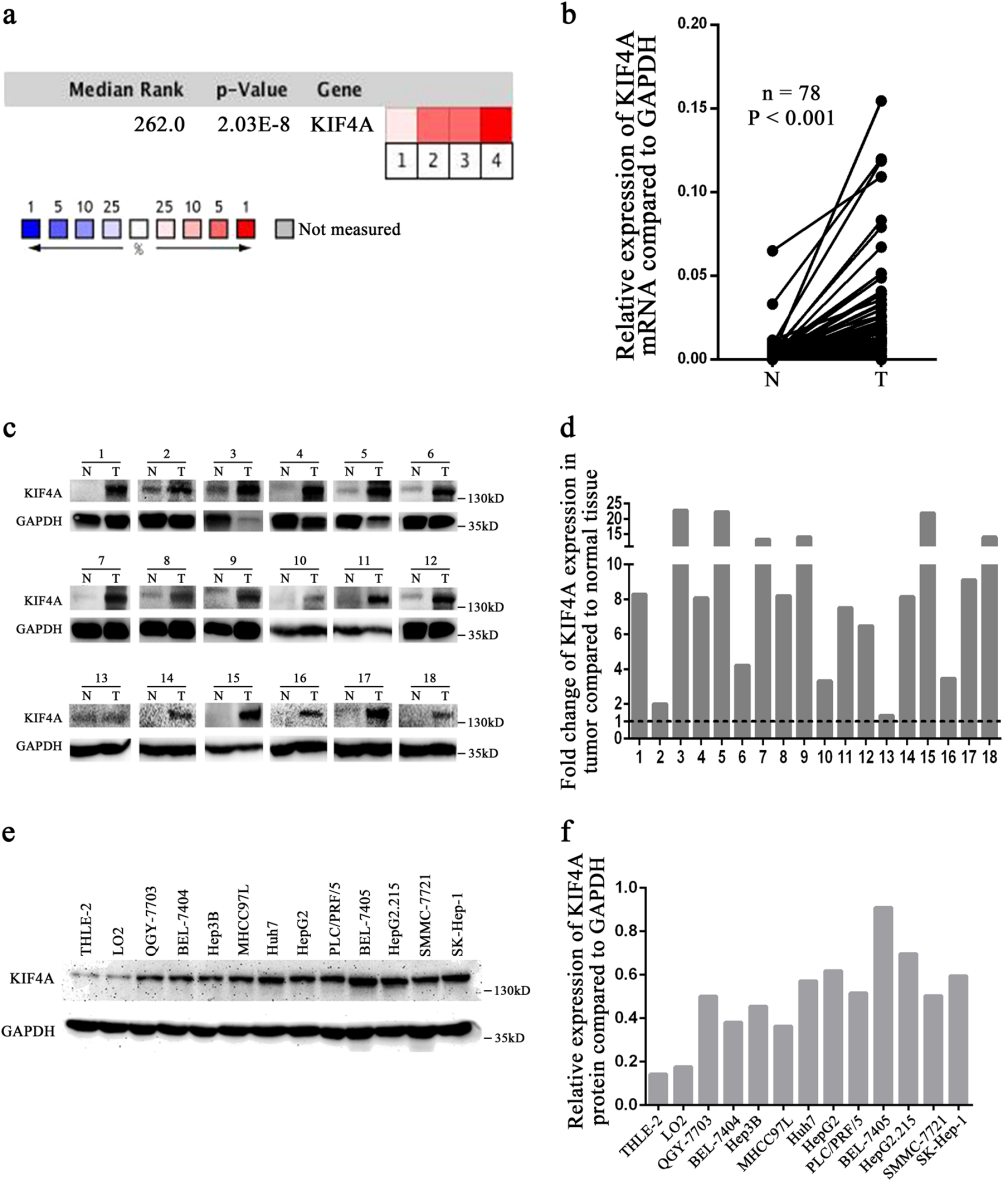
KIF4A expression increases in HCC tissues and cell lines **a** KIF4A mRNA expression in liver cancer tissues is higher than that in normal tissues from Oncomine database. Dataset 1, Wurmbach Liver; Dataset 2, Roessler Liver 1; Dataset 3, Roessler Liver 2; Dataset 4, Mas Liver. Cell colour and the number above the cell in the lower panel indicate the best gene rank percentile for the analysis. Red, upregulated; blue, downregulated. **b** The mRNA levels of KIF4A from 78 patients were tested by quantitative PCR using paired *t*-test. **c** The protein levels of KIF4A in HCC tissues and matched non-cancerous tissues from 18 patients with HCC were determined by western blotting assay. N non-cancerous, C cancer. Fold change of KIF4A protein with respect to non-cancerous specimens was normalized to GAPDH. The quantification of western blotting is shown in (**d**). **e** KIF4A protein expressions in nine HCC cell lines (QGY-7703, BEL-7404, Hepa3B, MHCC-97L, Huh7, PLC/PRF/5, BEL-7405, SMMC-7721, SK-HEP-1), two hepatoblastoma cell lines (HepG2, HepG2.215), and two immortalized liver cell lines (THLE-2 and LO2) were examined by western blotting. **f** Quantification of KIF4A expressions in different cell lines is shown

To assess the role of KIF4A in HCC, we analysed four microarray datasets from Oncomine database, and found significant overexpression of KIF4A in the majority of HCC tissues compared with adjacent non-neoplastic controls (Supplementary Fig. S1). The median rank of KIF4A in upregulated genes of HCC was 262.0, based on a meta-analysis across four datasets using the Oncomine algorithms^17^ (678 samples, *P* = 2.03E−8, Fig. 1a). To further validate the relationship between KIF4A expression status and HCC, we detected the mRNA level of tumour tissues compared with paired non-cancerous tissues using qPCR. Data confirmed that KIF4A mRNA levels were significantly elevated in tumour tissues (72/78, 92.3%, Fig. 1b). We also detected the KIF4A protein levels in 18 paired primary HCC tissues and the corresponding adjacent normal tissues using western blotting. Results showed that KIF4A protein levels (18/18,100%) were increased in primary liver cancer tissues compared with adjacent normal liver tissues (Fig. 1c, d). To further explore the potential role of KIF4A in HCC tumorigenesis, we detected the protein expression of KIF4A in 2 normal liver and 11 different HCC cell lines using western blotting. Compared with the normal liver cell lines THLE-2 and LO2, KIF4A was overexpressed in all the detected HCC cell lines (Fig. 1e, f). Taken together, these data indicated that KIF4A expression was increased in HCC tumour tissues and cells.

### Upregulation of KIF4A is associated with poor prognosis in liver cancer

**Fig. 2:**
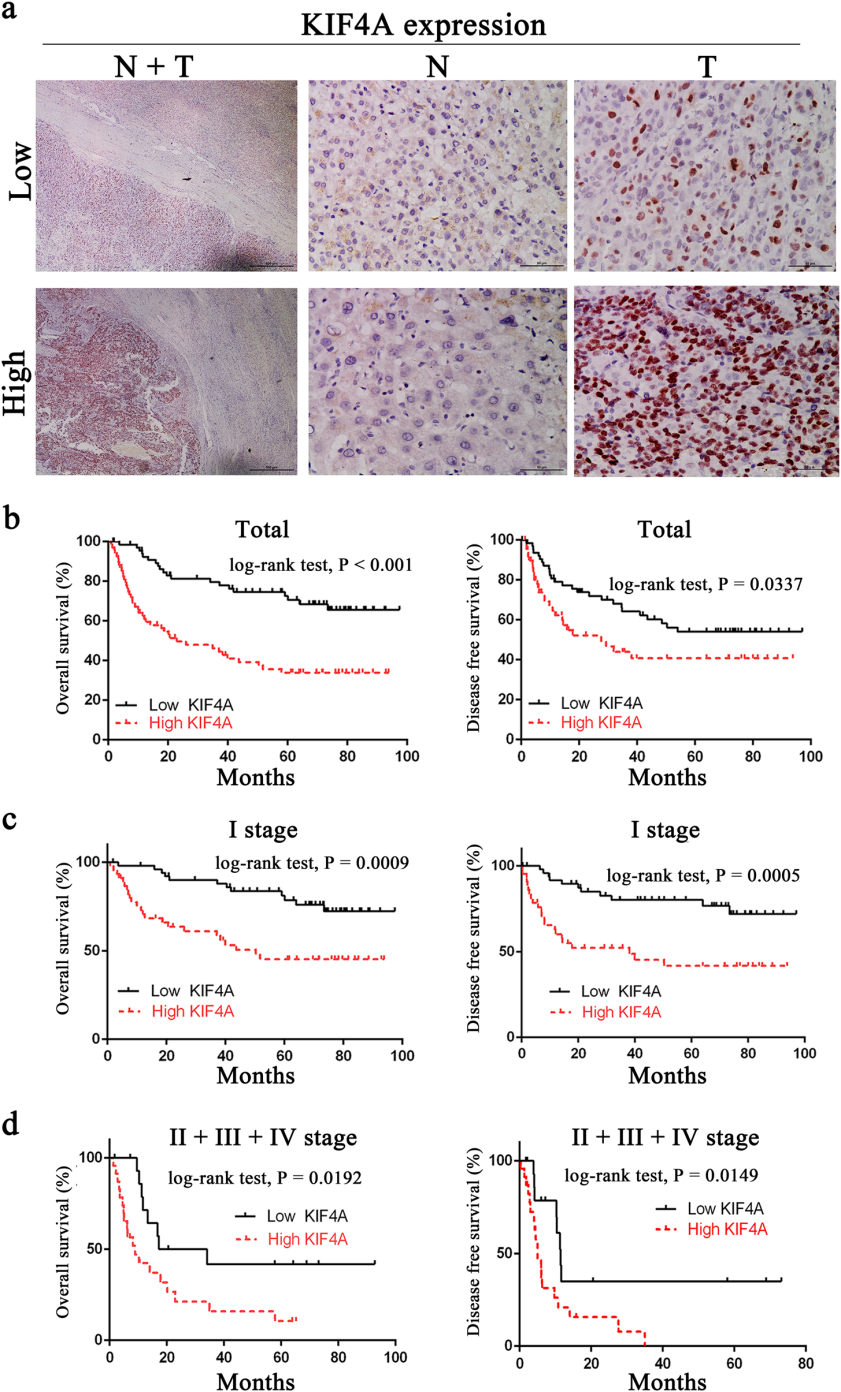
Upregulation of KIF4A is associated with poor prognosis in liver cancer **a** Immunohistochemical staining of KIF4A protein expression in 136 HCCs and their corresponding non-cancerous tissues. Two representative cases were shown. The scale bar of the left panel is 500 μm. The scale bar of the middle and right panels is 50 μm. **b** KIF4A expression was associated with OS (*n* = 136, *P* < 0.001) and DFS (*n* = 136, *P* < 0.001) according to Kaplan–Meier analysis. **c** Subgroup analysis for OS (*P* = 0.009) and DFS (*P* = 0.005) of HCC patients in TNM stage I. **d** Subgroup analysis for OS (*P* = 0.0192) and DFS (*P* = 0.0149) of HCC patients in TNM stage II + III + IV

To further investigate the KIF4A expression in fresh HCC tissues, we employed IHC staining with a total of 136 HCC samples from Sun Yat-sen University Cancer Center after liver section and followed up for 100 months. Among the patients, there were 18 women (13.2%) and 118 men (86.83%) with an age range from 20 to 78 years. Most patients (81.6%) had a single tumour, and in the majority the tumour size was >3 cm (118/136 cases, 86.7%). In the case of histological classification, 97 cases (71.3%) were classified as TNM stage I, 6 cases (4.4%) as TNM stage II, 22 cases (16.2%) as TNM stage III, and 11 cases (8.1%) as TNM stage IV. We identified that KIF4A expression was specifically detected in the nucleus of HCC cell from the cancer tissues in the vast majority of HCC samples (122/136 cases, 89.7%), while others presented negative expression (14/136 cases, 10.3%). No signals were found in all the corresponding adjacent normal tissues (Supplementary Figure S2). According to the IHC results, 68 cases were defined as low KIF4A expression, while the other 68 cases were identified as high expression (Fig. 2a). Further investigation of the relationship between KIF4A protein expression and the clinicopathological features is summarized in Table 1. Results indicated that KIF4A protein expression levels were markedly correlated with encapsulation (*P* = 0.036), tumour size (*P* = 0.004), and survival (*P* = 0.0003).

**Table 1:** Association of KIF4A expression with clinicopathological parameters in 136 HCC specimens

Parameters	Total	KIF4A expression		*P* value
		Low	High	
Gender				
Female	18	6	12	0.205
Male	118	62	56	
Age (years)				
≤50	85	43	42	1
>50	51	25	26	
Encapsulation				
Yes	74	31	43	0.036*
No	59	36	23	
Tumour size (cm)				
≤3	18	15	3	0.004*
>3	115	52	63	
Tumour number				
Single	87	58	49	0.122
Multiple	25	9	16	
Metastasis				
Yes	121	62	59	0.561
No	12	5	7	
Cirrhosis				
Negative	29	18	11	0.398
Positive	104	49	55	
Thrombosis				
Yes	13	6	7	1
No	123	62	61	
Differentiation grade				
Well	6	2	4	0.288
Middle	120	59	61	
Poor	5	4	1	
Ascites				
Yes	12	6	6	1
No	124	62	62	
HBsAg				
Negative	17	11	6	0.3
Positive	115	56	59	
HBeAg				
Negative	124	63	61	1
Positive	8	4	4	
AFP (ng/ml)				
≤400	70	39	31	0.295
>400	62	28	34	
PT (s)				
≤14	101	50	51	0.683
>14	31	17	14	
PLT (10^9^/L)				
≤100	9	5	4	1
>100	123	62	61	
ALT (U)				
≤40	68	36	32	0.604
>40	65	31	34	
AST (U)				
≤40	59	37	22	0.015*
>40	74	30	44	
Albumin (g/L)				
≤40	42	20	22	0.712
>40	91	47	44	
Bilirubin (μmol/L)				
≤17.1	72	40	32	0.225
>17.1	61	27	34	
TNM stage				
I	97	52	45	0.225
II+III+IV	39	16	23	
Recurrence				
Yes	78	40	38	0.86
No	53	26	27	
Survival				
Died	62	20	42	0.0003*
Alive	74	48	26	

Statistical analysis was performed by the Pearson *χ*^2^-test

*HCC* hepatocellular carcinoma, *HBsAg* hepatitis B surface antigen, *HBeAg* hepatitis B e antigen, *AFP* alpha-fetoprotein, *PT* prothrombin time, *PLT* platelet, *ALT* alanine transaminase, *AST* aspartate transaminase

*Represent *P* values with significant difference

According to Kaplan–Meier survival analysis, patients with higher KIF4A expression predicted a decreased OS (overall survival, *P* < 0.0001) and DFS (disease-free survival, *P* < 0.0001, Fig. 2b). When we stratified the data based on the TNM stage, higher KIF4A levels were found, suggesting a poorer OS (*P* = 0.0009) and DFS (*P* = 0.0005) in TNM stage I group (Fig. 2c). Consistent results showed that in the TNM stage II + III + IV group, higher KIF4A expression also was accompanied by poorer OS (*P* = 0.0192) and DFS (*P* = 0.0149, Fig. 2d). Multivariate Cox regression analysis showed that KIF4A expression (HR = 1.147, *P* = 0.001), age (HR = 2.265, *P* = 0.0336), AFP (HR = 1, *P* = 0.004), AST (HR = 1.025, *P* < 0.001), bilirubin (HR = 1.069, *P* = 0.006), HCC differentiation (HR = 0.321, *P* = 0.009) and TNM stage (HR = 2.043, *P* < 0.001) were independent predictors of survival in HCC patients (Table 2). These data indicated that KIF4A expression was associated with certain clinicopathological factors and could be a prognostic marker for both early- and late-stage HCC patients.

**Table 2:** Univariate and multivariate analysis of overall survival in 136 HCC specimens

Variables	Univariate analysis			Multivariate analysis		
	Hazard ratio	95% CI	*P* value	Hazard ratio	95% CI	*P* value
Gender	1.242	0.796–2.006	0.375			
Age (years)	1.003	0.988–1.019	0.689	2.265	1.064–6.188	0.036*
Encapsulation	1.714	1.266–2.319	<0.001*			
Tumour size (cm)	1.149	1.097–1.203	<0.001*			
Tumour number	2.683	1.809–3.979	<0.001*			
Metastasis	3.143	1.587–6.225	<0.001*			
Cirrhosis	1.881	1.139–3.107	0.014*	2.253	0.981–5.174	0.056
Thrombosis	4.425	2.687–7.286	<0.001*			
Differentiation grade	0.904	0.414–1.973	0.8			
Ascites	1.746	1.032–2.955	0.038*			
HBsAg	0.961	0.550–1.681	0.889			
HBeAg	1.685	0.674–4.125	0.265			
AFP (ng/ml)	1	1.000–1.000	<0.001*	1	1.000–1.000	0.004*
PT (s)	0.977	0.941–1.014	0.214			
PLT (10^9^/L)	1.002	1.000–1.005	0.107			
ALT (U)	1.009	1.000–1.017	0.042*			
AST (U)	1.029	1.021–1.037	<0.001*	1.025	1.011–1.038	<0.001*
Albumin (g/L)	0.946	0.909–0.984	0.006*			
Bilirubin (μmol/L)	1.035	1.008–1.063	0.012*	1.069	1.019–1.121	0.006*
TNM stage	1.698	1.469–1.964	<0.001*	2.043	1.568–2.637	<0.001*
Recurrence	2.111	1.264–3.542	0.004*			
KIF4A scores	1.159	1.105–1.241	<0.001*	1.147	1.061–1.240	0.001*

Statistical analysis was performed by Cox test analysis

*HCC* hepatocellular carcinoma, *HBsAg* hepatitis B surface antigen, *HBeAg* hepatitis B e antigen, *PT* prothrombin time, *PLT* platelet, *ALT* alanine transaminase, *AST* aspartate transaminase

*Represent *P* values with significant difference

### KIF4A promotes proliferation and clonogenicity of HCC cells

**Fig. 3:**
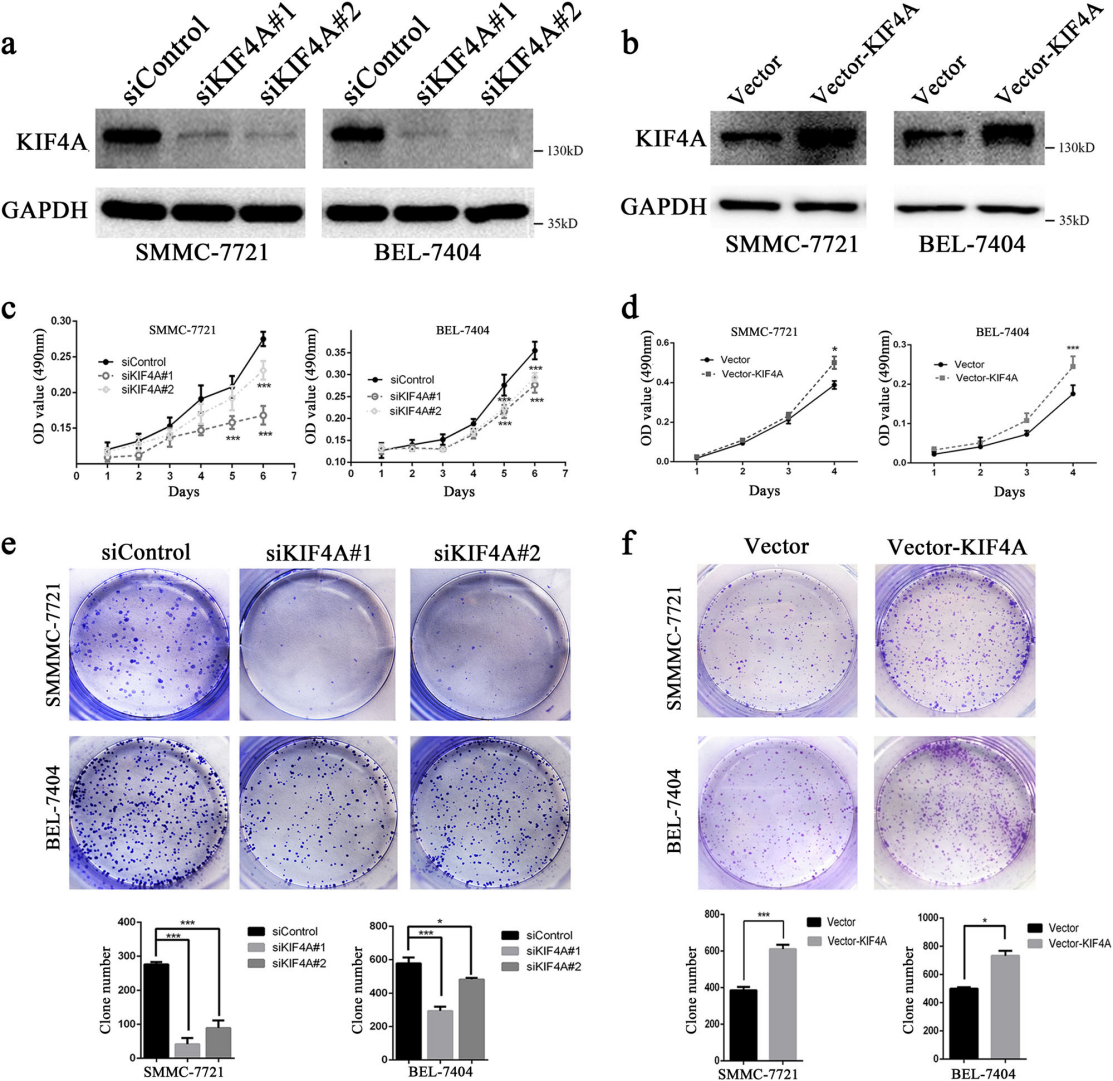
KIF4A promotes proliferation and clonogenicity of HCC cells **a** The effect of KIF4A knockdown with siRNAs was verified by western blotting 72 h after transfection. **b** The effect of KIF4A overexpression was verified by western blotting. **c** Viability of KIF4A knockdown cells was assessed with an MTT assay at the indicated times. **d** Viability of KIF4A overexpression cells was assessed with an MTT assay at the indicated times. **e** Colony formation assays of SMMC-7721 and BEL-7404 cells transfected with negative control and KIF4A-targeted siRNAs. Upper panel: representative image, lower panel: quantification of the colony numbers. **f** Colony formation assays of control and KIF4A-overexpressing HCC cells. Upper panel: representative image, lower panel: quantification of the colony numbers. Statistically significant difference: *P < 0.05, **P < 0.01, ***P < 0.001

To address the potential role of KIF4A in HCC progression, KIF4A knockdown and overexpression of HCC cell models were constructed in SMMC-7721 and BEL-7404 cells with two distinct siRNA duplexes and the lentivirus infection method, respectively. As shown in Fig. 3, KIF4A expression was almost eliminated in knockdown cell models (Fig. 3a) and increased in overexpressing cell models, indicating successful establishment (Fig. 3b). MTT assay was then performed to assess cell viability at the indicated times. Data showed that the inhibition of KIF4A markedly declined the HCC cells' viability (Fig. 3c). On the contrary, cellular proliferation ability greatly increased after KIF4A overexpression (Fig. 3d). Colony formation assay showed that, compared with the siNC cells, both the size and number of siKIF4A transfectants were dramatically decreased (Fig. 3e). On the other hand, the size and number were significantly increased in KIF4A-overexpressing cells (Fig. 3f). We also investigated the proliferation-related marker Ki67 in 53 fresh HCC tissues by immunohistochemistry (IHC) (Supplementary Fig. S3a). The results suggested that there was a significant positive correlation between expressions of KIF4A and Ki67 (Supplementary Figure S3,b). Taken together, these results indicated that KIF4A played an important role in HCC proliferation and clonogenicity.

### KIF4A is required for proper mitosis maintenance

**Fig. 4:**
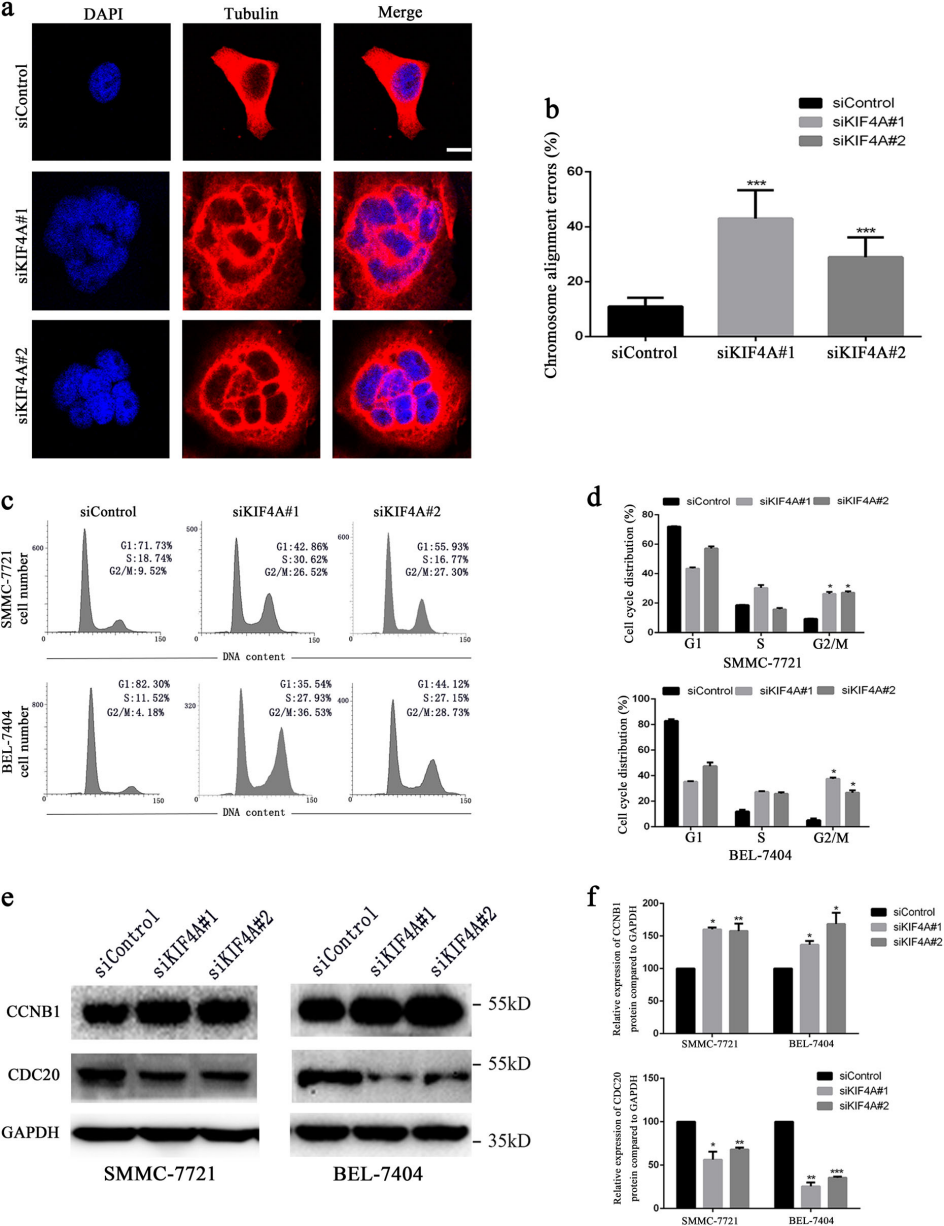
KIF4A is required for proper mitosis maintenance **a** SMMC-7721 cells were transfected with control or KIF4A siRNAs. Forty-eight hours after transfection, cells were fixed and stained with anti-tubulin (red) antibody and DAPI (blue) and visualized under a confocal microscope. Scale bar = 10 μm. Quantification of cells with mitotic defects was shown in (**b**). Representative images of cell cycle distributions of SMMC-7721 and BEL-7404 cells transfected with control or KIF4A siRNAs for 48 h were determined by flow cytometry (**c**). Flow cytometry results are summarized in (**d**). Results are representative of three independent experiments performed in triplicate. The data are presented as the means ± SD. Cells treated with siKIF4A showed downregulation of CDC20 and upregulation of cyclin B1 compared with control cells (**e**). The quantification for the blots is shown in (**f**). The data are presented as the means ± SD. Statistically significant difference: *P < 0.05, **P < 0.01, ***P < 0.001

To disclose the underlying mechanism responsible for KIF4A-mediated HCC cell proliferation and clonogenicity, the effect of KIF4A knockdown was further evaluated in SMMC-7721 cells. We first observed that through immunofluorescence staining the number of multinucleated cells increased after siKIF4A treatment, suggesting that KIF4A knockdown might affect chromosome misalignment and mitosis (Fig. 4a, b). We further investigated whether KIF4A depletion could cause cell cycle arrest. SMMC-7721 and BEL-7404 were synchronized at G1/S transition by double thymidine block and then released to fresh media to continue the cell cycle process. We harvested the cells and analysed their cell cycle distribution at the indicated time points. Results showed that the fraction of cells in G2/M phase was significantly increased in siKIF4A transfectants, indicating that KIF4A knockdown can trigger the G2/M phase arrest in both SMMC-7721 and BEL-7404 cells (Fig. 4c, d). According to the previous study on oral cancer, KIF4A depletion contributes to activating the SAC during cell division^13^. SAC monitors the attachment of chromosome to the mitotic spindle and allows the chromosome separates precisely, and it is an inhibitor of the anaphase-promoting complex or cyclosome (APC/C) and CDC20. The APC/C, a major ubiquitin ligase activated by CDC20, regulates the exact timing of cyclin B degradation to trigger anaphase onset. When chromosomal misalignment occurs, degradation of cyclin B1 is inhibited^18^. Consistent with the above research, we measured the expression level.s of CDC20 and cyclin B1 in KIF4A knockdown cells and found that the expression of CDC20 was significantly downregulated, while cyclin B1 was upregulated (Fig. 4e, f). In summary, these data suggested that KIF4A might be essential for proper mitotic progression by precisely orchestrating chromosome alignment and segregation.

### KIF4A maintains cell survival via activation of PI3K/Akt pathway

**Fig. 5:**
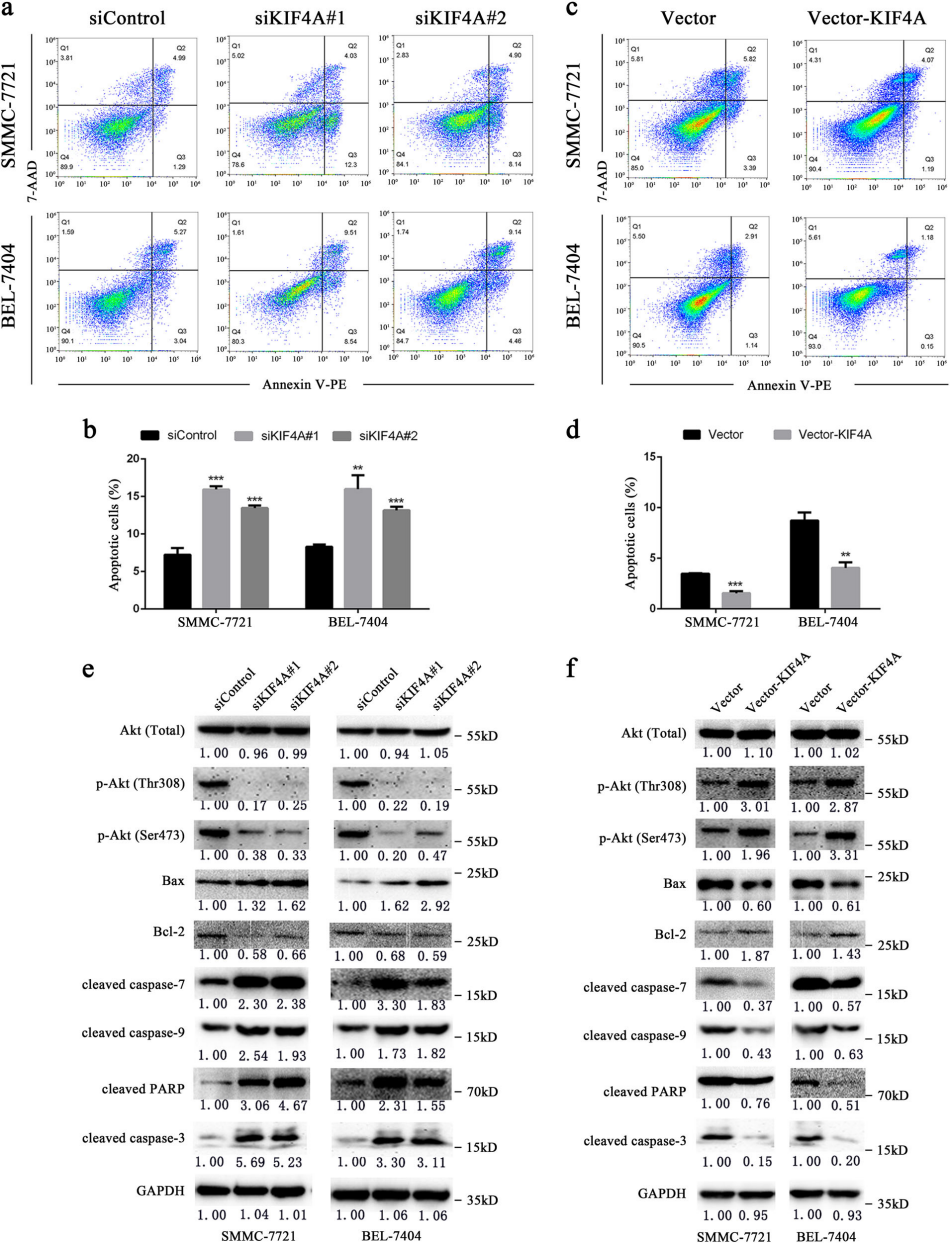
KIF4A maintains cell survival via activation of the PI3K/Akt pathway **a**,** b** Representative images of apoptosis analysis by flow cytometry in SMMC-7721 and BEL-7404 cells after KIF4A depletion (**a**), or overexpression (**b**). **c**, **d** Quantifications of apoptotic cells in SMMC-7721 and BEL-7404 cells after KIF4A depletion (c), or overexpression (**d**). **e**,**f** Western blotting analysis of expression of total Akt, p-Akt (Thr308), p-Akt (Ser408), Bax, Bcl-2, cleaved-PARP, cleaved-caspase-7, and cleaved-caspase-3 in SMMC-7721 and BEL-7404 cells after KIF4A depletion (**e**) or overexpression (**f**). Fold changes by densitometry normalized to controls are shown below. Statistically significant difference: *P  0.05, **P  0.01, ***P  0.001

Incomplete and aberrant mitosis often leads to cell apoptosis. Since we observed that KIF4A depletion caused abnormal mitotic progression, we measured the relationship of KIF4A regulation and cell apoptosis through Annexin V-FITC/PI dual staining assay. Flow cytometry analysis showed that KIF4A depletion increased the percentage of apoptotic cells (Fig. 5a, b), while apoptotic rates decreased significantly in KIF4A-overexpressing cell lines (Fig. 5c, d). According to a currently published study, KIF4A knockdown decreased the expression of p-Akt^19^. We speculated that KIF4A may contribute to maintaining the cell survival by regulating the PI3K/Akt pathway in our models. Western blotting results showed that protein levels of p-Akt (Ser473) and p-Akt (Thr308) were downregulated dramatically in the protein lysate of siKIF4A transfectants, while the total amount of Akt remained unchanged. Expression of Bax, an important pro-apoptosis factor downstream of Akt, was dramatically upregulated and anti-apoptosis factor Bcl-2 was downregulated. Most importantly, we found that cellular apoptosis markers such as cleaved-caspase-3, cleaved-caspase-7, and cleaved-PARP were significantly upregulated after KIF4A depletion (Fig. 5e). Similarly, we accessed the expression of the above proteins in KIF4A-overexpressing cell lines, which had been cultured without serum for 48 h. Compared with control cells, total Akt expression was unchanged, p-Akt (Ser473) and p-Akt (Thr 308) were dramatically upregulated, Bcl-2 was upregulated, and Bax was downregulated. Apoptosis markers including cleaved-caspase-3, cleaved-caspase-7, and cleaved-PARP were downregulated significantly in KIF4A-overexpressing cell lines (Fig. 5f). These results indicated that KIF4A maintained cell survival by activating the Akt signaling pathway.

### Skp2 correlates positively with KIF4A expression in HCC

**Fig. 6:**
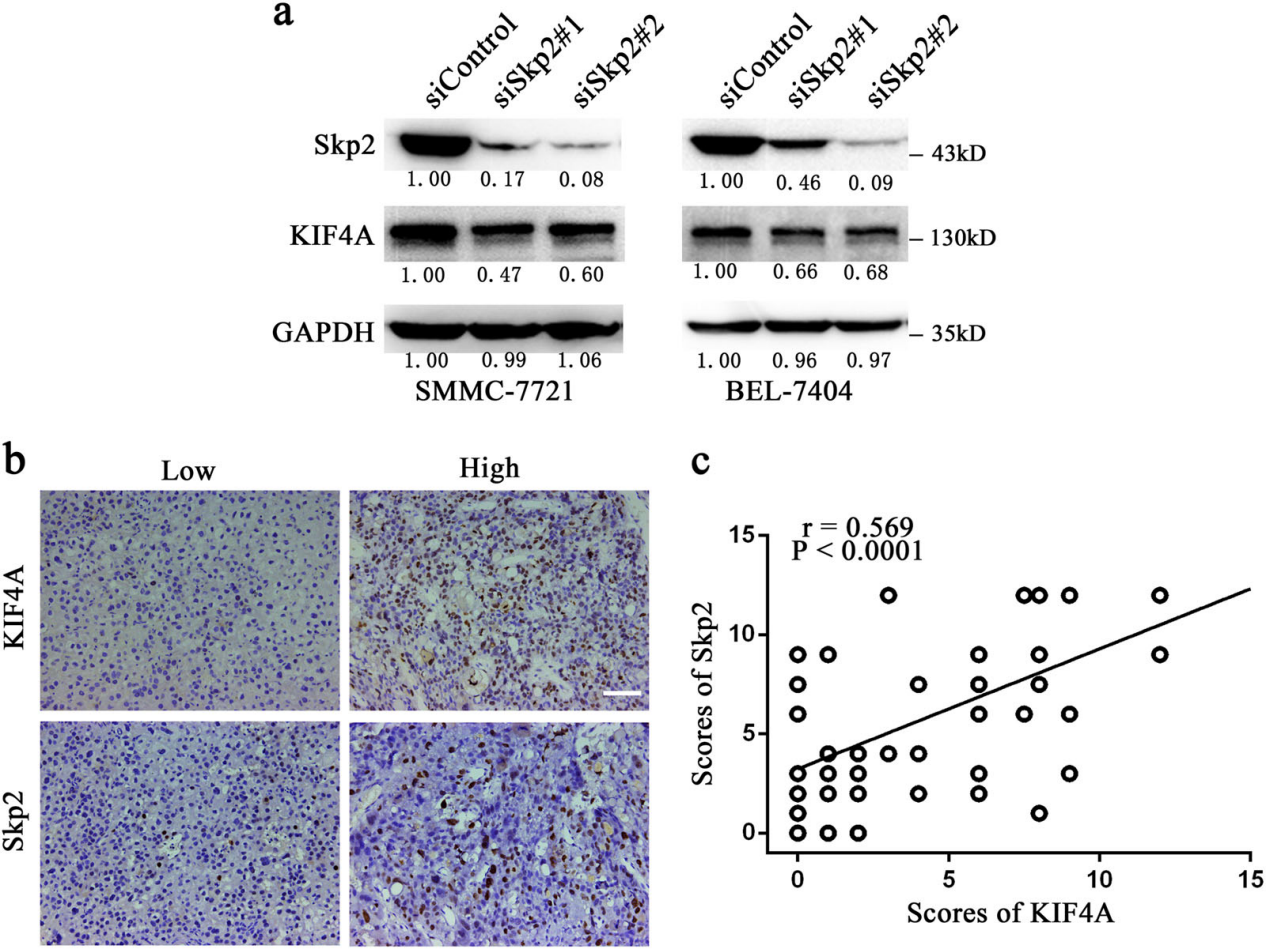
Skp2 regulates the expression of KIF4A **a** Expression levels of Skp2 and KIF4A were detected by western blotting in SMMC-7721 and BEL-7404 cells transfected with Skp2 and control siRNAs. Fold changes by densitometry normalized to controls are shown below. **b** Immunohistochemical staining of KIF4A and Skp2 protein expression levels in 53 HCC tissues. Representative images are shown. Scale bar 100 μm. **c** Scatterplot of immunoreactivity scores of Skp2 vs. KIF4A with regression line showed a positive correlation

Skp2 is a component of the SCF^Skp2^ ubiquitin E3 ligase complex, and responsible for recruiting substrate proteins and subsequent ubiquitin-proteasome-dependent degradation^20^. Overexpression of Skp2 is well known for its strong association with aggressive tumour behaviour and poor clinical outcome in a variety of cancers, including HCC^21^. Recently, Xu et al.^22^ reported that KIF14, a structurally and functionally similar protein to KIF4A, and also a member of the kinesin superfamily of proteins^23^, regulated the expression of Skp2 in HCC through an undefined mechanism. Due to its significant role in cancer development and progression, we wondered whether Skp2 expression was also related to KIF4A in this study. We silenced Skp2 with siRNAs in SMMC-7721 and BEL-7404 cells. Effective knockdown of Skp2 led to significantly decline of KIF4A expression in HCC cells (Fig. 6a). Furthermore, we performed immunohistochemical staining of Skp2 and KIF4A in 53 HCC samples, graded, and performed correlation analysis. We found that Skp2 showed a significant positive correlation with KIF4A in HCC tissues (Fig. 6b, c). These results suggested that expression levels of Skp2 and KIF4A correlated positively with each other in HCC.

## Discussion

HCC is characterized by multiple cancer hallmarks, including genetic and epigenetic alterations that cause uncontrolled cellular proliferation and cell cycle regulation. In recent years, there has been a surging interest in studying the novel genes that are involved in cancer development and progression. Here, we demonstrated that KIF4A is overexpressed in HCC tissues and cell lines, and KIF4A overexpression predicts a poor prognosis for HCC patients. We then used in vitro HCC cell models to address the molecular mechanism by which KIF4A promotes hepatic malignant transformation and tumour progression. Through loss-of-function study we found that KIF4A depletion induces G2/M phase arrest and suppresses mitotic progression. Moreover, we showed that KIF4A depletion induces apoptosis by inhibiting Akt kinase activity in HCC cells. In addition, Skp2 knockdown decreases KIF4A expression and their expression levels show a positive correlation in HCC tissues. Thus, this study presents a key role of KIF4A in promoting cellular growth and maintaining normal mitotic progression in HCC.

Similar to other investigations of rapid growth rate in HCC, KIF4A overexpression enhances proliferation and colony formation abilities in HCC cells, highlighting its importance in HCC progression. KIF4A participates in chromosome condensation and segregation in multiple steps during the process of mitotic division by acting as an essential component in regulating the completion of cytokinesis and anaphase spindle dynamics^24^. As a matter of fact, KIF4A depletion may cause defects in mitotic chromosome formation and subsequent mitotic checkpoint activation, resulting in uncompleted cytokinesis. In line with this expectation, a published study in oral cancer cells demonstrated that KIF4A knockdown may lead to SAC activation, which finally causes the G2/M arrest^13^. Consistent with these studies, we also observed that, after KIF4A depletion, a large amount of HCC cells were arrested in G2/M phase and became multinucleated. Considering its conserved role in cytokinesis, it is likely that KIF4A supported HCC cell growth by a similar mechanism that maintains proper chromosome architecture during mitosis. Therefore, we can speculate that KIF4A overexpression possibly contributes to uncontrolled cell cycle progression and division in hepatocytes, which may cause HCC initiation and development.

Apoptosis is a genetically regulated, cellular suicide mechanism that plays a crucial role in maintenance of physiological homeostasis and development. There are two typical apoptosis signalling pathways, namely extrinsic and intrinsic pathways, which converge on the effector molecules caspase-3 and -7 to cleave the downstream targets and induce apoptotic phenotype. Our data showed that KIF4A knockdown resulted in decline of Bcl-2 expression, increase of Bax expression and cleavage of caspase-9, which are mediators of the intrinsic apoptosis pathway. Disassociation of Bcl-2 with Bax is important to trigger intrinsic apoptosis cascade by modulating mitochondria function^25^. On the basis of these observations, we suggested that KIF4A depletion might inhibit HCC cell proliferation through the mitochondria apoptosis pathway. Moreover, activation of Akt is sufficient to block the release of cytochrome c by directly phosphorylating Bax and suppressing its translocation to the mitochondria membrane^26^, and a recent study reported that silencing KIF4A inhibited the activation of Akt^19^. Therefore, we tried to define whether KIF4A would regulate Bax expression through the Akt signalling pathway. In compliance with the above research, our study showed that KIF4A knockdown suppressed the phosphorylation of Akt, along with a higher expression of Bax protein. Contradicting results were obtained using the KIF4A-overexpressing cell models. Therefore, these results suggest that KIF4A may be involved in the intrinsic pathway and may protect cells from apoptosis by activating the PI3K/Akt pathway. However, the exact mechanism needs further characterization.

Worldwide, China has been recognized as an area with a significantly high incidence of HBV infection. Evidence shows that HBV-related cancer development and poor prognosis are independently associated with several viral factors, such as HBV DNA, HBV genotype C, and HBV core promoter mutations^27–29^. The risk of HCC development in patients with chronic HBV infection is 100 times greater than in healthy controls^30^. Our previous studies showed that mutations in HBV genome mutations upregulate Skp2 expression, leading to increased risk of HCC^5,6^. In this study, we demonstrated that Skp2 depletion resulted in KIF4A downregulation, and their expression correlated with each other in our HCC samples. Considering our HCC patients have a nearly 90% rate of HBV infection, we wondered if HBV infection would regulate KIF4A expression in HCC. In fact, a recent study reported that HBV activated the KIF4A gene promoter and upregulated the mRNA and protein expression levels of KIF4A in HCC cell lines^31^. However, further investigations are needed to clarify the underlying mechanism how HBV regulates KIF4A expression.

Our findings are meaningful for the following reasons. First, the scale of HCC samples is large, which could better demonstrate the result that KIF4A overexpression is associated with poor prognosis in HCC. Second, many studies have assessed clinicopathological factors based on 3-years survival, whereas we demonstrated that KIF4A exerted an additive effect over a longer period with the 8-years survival of patients with HCC. Third, it is the first time to demonstrate that knockdown of KIF4A could induce G2/M arrest and promote apoptosis in HCC cells. Fourth, we proposed that HBV may be involved in KIF4A regulation through a Skp2-mediated mechanism. However, our study also has limitations in that animal experiments are needed to validate KIF4A’s function in vivo and further investigations are awaited to explain the exact molecular mechanism behind association of Skp2 and KIF4A expression.

In conclusion, we demonstrated that KIF4A is overexpressed in HCC tissues and cell lines. Higher level of KIF4A in HCC patients predicts a poor prognosis. KIF4A depletion impairs cellular proliferation and colony formation abilities in HCC cells. Additionally, KIF4A expression is required for the maintenance of normal mitotic progression and protection from apoptosis in HCC cells. Taken together, KIF4A may act as a prognostic biomarker and potential therapeutic target in human HCC.

## Materials and methods

### Materials

The commercially available antibodies used are as follows: KIF4A (sc-365145,Santa Cruz), cleaved-caspase-3 (#9915, Cell Signaling Technology), cleaved-caspase-7 (#8438, Cell Signaling Technology), cleaved-poly ADP-ribose polymerase (PARP, #5625, Cell Signaling Technology), Bcl-2 (#4223, Cell Signaling Technology), Bax (#5023, Cell Signaling Technology), Akt (pan) (#4691, Cell Signaling Technology), p-Akt (ser473) (#4060, Cell Signaling Technology), p-Akt (Thr308) (#13038, Cell Signaling Technology) and Skp2 (#2652s, Cell Signaling Technology), CDC20 (10252-1-AP, Proteintech), cyclin B1 (#4138, Cell Signaling Technology), α-Tubulin (66031-1-Ig, Proteintech), GAPDH (60004-1-Ig, Proteintech) and Ki67 (MA5-14520, Rochford).

### Patient selection and tissue preparation

We obtained the paraffin-embedded HCC specimens (*n* = 136) for prognostic survival analysis from Sun Yat-sen University Cancer Center (Guangzhou, China). For analysing the association between KIF4A and Ki67, another 53 fresh HCC specimens were collected from the Third Affiliated Hospital of Sun Yat-sen University (Guangzhou, China). A surgical tumour resection was performed on each patient in the Department of Hepatobiliary Surgery. Then tissues were cut into proper size and stored in liquid nitrogen directly for RNA and protein extraction or fixed in 4% paraformaldehyde for IHC. The study was approved by the Institute Research Ethics Committee at the Sun Yat-sen University Cancer Center and the Third Affiliated Hospital of Sun Yat-sen University (Guangzhou, China). Written informed consent was obtained from each patient. Relative experiments with these samples were performed in accordance with the relevant regulations.

### Immunohistochemistry

IHC was performed as previously described^28^. Briefly, all paraffin-embedded HCC samples were cut into 4-μm sections on a glass slide. Then these slides were dried overnight at 37 ℃, deparaffinized in xylene twice for 10 min and rehydrated through graded alcohol five times for 5 min, immersed in 3% hydrogen peroxide for 15 min to block endogenous peroxidase. The sections were boiled in an electric pressure cooker in ethylenediamine tetraacetic acid (EDTA) buffer (pH = 8.0) to retrieve antigen for 3 min. Then, the slides were incubated with 10% normal goat serum at room temperature for 30 min to reduce nonspecific reaction. Sections were then incubated overnight with primary antibody against KIF4A, Ki67, or Skp2 at 4 ℃ and anti-rabbit/mouse secondary antibody at room temperature for 1 h. Signals were detected in a freshly prepared DAB substrate solution at room temperature for 5 min. Finally, the sections were counterstained with Mayer’s haematoxylin, dehydrated, and mounted. Each section was evaluated by three independent pathologists who were blinded to the clinical status of patients and graded as described, according to positive staining intensity (0 = no staining, 1 = weak staining, 2 = moderate staining, 3 = strong staining) and the expression extent scores (percentage of positive cells)^30^. A final immunoreactivity score (IRS) was defined as the intensity score multiplied by the extent score.

### Cell culture

Eleven HCC cell lines (QGY-7703, BEL-7404, Hepa3B, MHCC-97L, Huh7, HepG2, PLC/PRC/5, BEL-7405, HepaG2.2.15, SMMC-7721 and SK-HEP-1) and two immortalized liver cells (THLE-2 and LO2) were employed in this study, cultured in Dulbecco’s Modification of Eagle’s Medium (DMEM, Gibco, Carlsbad, CA, USA) containing 10% foetal bovine serum (FBS, Gibco) at 37 ℃ and 5% CO_2_. THLE-2 was purchased from American Type Culture Collection (Manassas, VA, USA). Others were obtained from the College of Life Science, Sun Yat-sen University (Guangzhou, China). Cells were digested and passaged regularly.

### Reverse transcription and quantitative PCR

Total RNA was isolated from tissue specimens and HCC cell lines using Trizol reagent (Invitrogen, Carlsbad, CA, USA) according to the manufacturer’s protocol. Total RNA (1 µg) was reverse transcribed into cDNA by the GoScript™ Reverse Transcription System (Promega). Quantitative PCR (qPCR) was performed in three duplicate wells by employing SYBR Green (Promega, USA) in Roche LightCycler 96 (Roche Applied Science, Penzberg, Germany). Specific primers were 5′-TACTGCGGTGGAGCAAGAAG-3′ (forward) and 5′-CATCTGCGCTTGACGGAGAG-3′ (reverse) for KIF4A, and 5′-GGAGCGAGATCCCTCCAAAAT-3′ (forward) and 5′-GGCTGTTGTCATACTTCTCATGG-3′ (reverse) for GAPDH.

### Western blotting

Western blot assay was performed as standard procedure. Total protein was extracted using Radio Immunoprecipitation assay (RIPA) buffer with protease/phosphatase inhibitor cocktail (Roche). Protein concentration was measured by BCA protein assay. Protein was then separated using 8–12% gradient polyacrylamide gel and then transferred onto polyvinylidene difluoride (PVDF) membranes. The membranes were blocked in Tris-buffered saline (TBS) containing 5% bovine serum albumin (BSA) at room temperature for 1 h and subsequently incubated with the indicated primary antibody at 4 ℃ overnight and then with the secondary antibody at room temperature for 1 h. Bands were visualized using the enhanced chemiluminescence (ECL, Pierce). Quantification of band densitometry was measured with ImageJ software.

### Plasmid construction and RNA interference

KIF4A coding sequence was amplified and inserted into LV003-IRES-EGFP (Forevergen Biosciences Co., Ltd). Lentiviruses were produced by co-transfecting constructed plasmids and packaging plasmids psPAX2 and pMD2.G (Addgene) into 293T using Lipofectamine 2000 (Invitrogen, Carlsbad, CA, USA) for about 72 h^32^. Culture supernatants were collected, filtered, concentrated and used to infect SMMC-7721 and BEL-7404. After 48 h of infection, infected cells were selected by 2 µg/mL puromycin (540411, Merck) and successful establishment was confirmed by western blotting.

Two targeting KIF4A siRNA duplexes (KIF4A RNA#1, 5′-GGAACAGGGCAACAACTCT-3′; KIF4A RNA#2, 5′-TGAGGATGGTGATGGTGAT-3′) were obtained from RiboBio company (Guangzhou, China) and gave consistent results. Two targeting Skp2 siRNA duplexes were referenced on a published study^33^. SMMC-7721 and BEL-7404 were transfected with 100 nM siRNA using Lipofectamine RNAiMAX according to the manufacturer’s protocol (Invitrogen, Carlsbad, CA, USA). Seventy-two hours later the RNA interference was confirmed using western blotting.

### MTT cell viability assay

Cell proliferation rate was determined using MTT assay (M6494, Thermo) according to the manufacturer’s protocol. Cells were seeded in five replicates in a 96-well plate at a density of 2000 cells per well and cultured with DMEM containing 10% FBS. For 7 days, cells were incubated with 20 μL of 5 mg/mL MTT for 4 h at 37 ℃. Subsequently, 150 μL of 100% dimethylsulphoxide (DMSO) was added to dissolve the precipitates. Viable cells were counted every day by reading the absorbance at 490 nm with a plate reader (BITELX800, BiTek).

### Colony formation assay

Thousand SMMC-7721 cells per well were plated in six-well plates and cultured in 37 ℃ for 14 days in DMEM with 10% FBS. In total 1500 BEL-7404 cells per well were seeded in six-well plates for 12 days. An additional culture medium was added to the plates on day 3. Cells were fixed with methanol, stained with 0.5% crystal violet (C6158, Sigma) and dried. The colony is defined to consist of at least 50 cells according to a previously described method^34^ and all the colonies were counted using a microscope. The test was repeated three times.

### Flow cytometry

For cell cycle analysis, cells were first synchronized at G1/S transition using double thymidine block as previously described^35^, and harvested at 16 h after release in fresh medium. Then samples were washed twice in PBS, and then fixed in ice-cold 70% ethanol at −20 °C overnight. Fixed cells were treated with RNase A (R4875, Sigma-Aldrich) for 30 min at room temperature before addition of 5 μL/mL propidium iodide (PI, P4864, Sigma-Aldrich) for 10 min in the dark and analysed by flow cytometry.

For apoptosis analysis, cells were stained with annexin V-PE and 7-AAD (AP104, Multi Sciences) and evaluated for apoptosis by flow cytometry according to the manufacturer’s protocol. Briefly, 1 × 10^6^ cells were washed twice with PBS and stained with 5 μL annexin V-PE and 10 μL 7-AAD in 1 × binding buffer for 15 min at room temperature in the dark. Apoptotic cells were determined using a Beckman-Coulter Flow Cytometry FC500. Both early (annexin V-positive/7-AAD-negative) and late (annexin V-positive/7-AAD-positive) apoptotic cells were included when assessing cell death.

### Immunofluorescence analysis

Cells were plated on chamber slides, fixed with 4% paraformaldehyde at 37 ℃ for 5 min. To keep the stability of microtubule capture at kinetochores, cells were incubated for 5 min on ice before fixation, to destabilize most non-kinetochore microtubules. After fixation, cells were permeabilized with 0.1% triton for 5 min. Then cells were blocked with 5% BSA for 20 min and incubated with the indicated primary antibodies at 4 ℃ overnight. The fluorescence-visualized secondary antibody was added and incubated for 60 min. Nucleus was stained with 50 ng/ml DAPI (4′,6-diamidino-2-phenylindole, D21490, Invitrogen) for 5 min at room temperature. Fluorescence signal was imaged using confocal microscope (LSM710, Zeiss). Multinucleated cells were defined as cells that have two or more nucleus per cell. The proportion of chromosome alignment errors was calculated as the ratio of multinucleated to total cells. At least 500 cells were counted for each group.

### Oncomine data analysis

Oncomine (http://www.oncomine.com) is an integrated cancer microarray database that contains unified bio-informatics resources from 715 datasets (version 4.4.4.3 after Q2 update 2013)^36^. We compared the mRNA expression of KIF4A from liver cancer datasets that contain data from both HCC tissues and normal liver tissues. Four datasets were included in our study: Wurmbach et al.^37^, Roessler et al (including Roessler Liver 1 and 2 datasets)^38^, and Mas et al.^39^. The differentiated expression for KIF4A between HCC tissues and normal liver tissues was analysed by *t*-test and their fold-change values and statistical significance determined by *P*-value were collected.

### Statistical analysis

A paired *t*-test was used to analyse the different mRNA levels of KIF4A in HCC tissues and matched adjacent tissues. Independent *t*-test was applied to analyse differences between two groups. A chi-squared test was employed to analyse the relationship between KIF4A expression and clinicopathological characteristics. The Kaplan–Meier analysis was employed for the survival analysis. The Spearman’s correlation coefficient was employed for KIF4A and Skp2 correlation analysis. All of the statistical tests were two-sided. Difference with *P* < 0.05 was considered statistically significant. All the statistical tests were performed using SPSS 20.0 statistical software (SPSS Company, Chicago, IL, USA).

## Electronic supplementary material

Revised Supplementary Figure S1

Revised Supplementary Figure S2

Revised Supplementary Figure S3

Supplementary Figure Legends
