# Supplementary figures and images for: Upregulation of kinesin family member 4A enhanced cell proliferation via activation of Akt signaling and predicted a poor prognosis in hepatocellular carcinoma
Source: Cell Death Dis. 2018 Feb 2;9(2):141. doi: 10.1038/s41419-017-0114-4 (PMC5833581; doi:10.1038/s41419-017-0114-4)

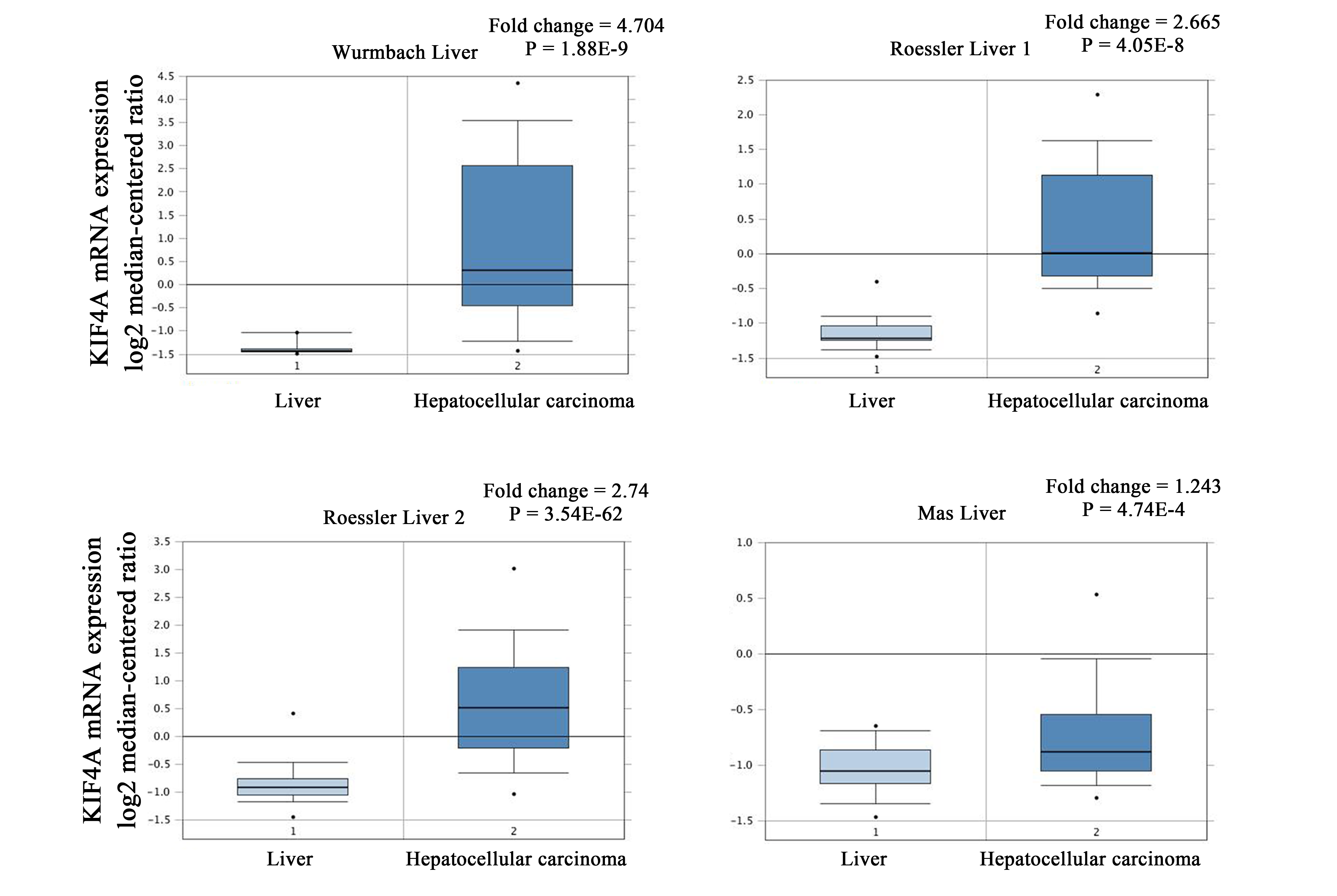

Supplement: Supplementary file 1 — Revised Supplementary Figure S1 [file 41419_2017_114_MOESM1_ESM.tif]

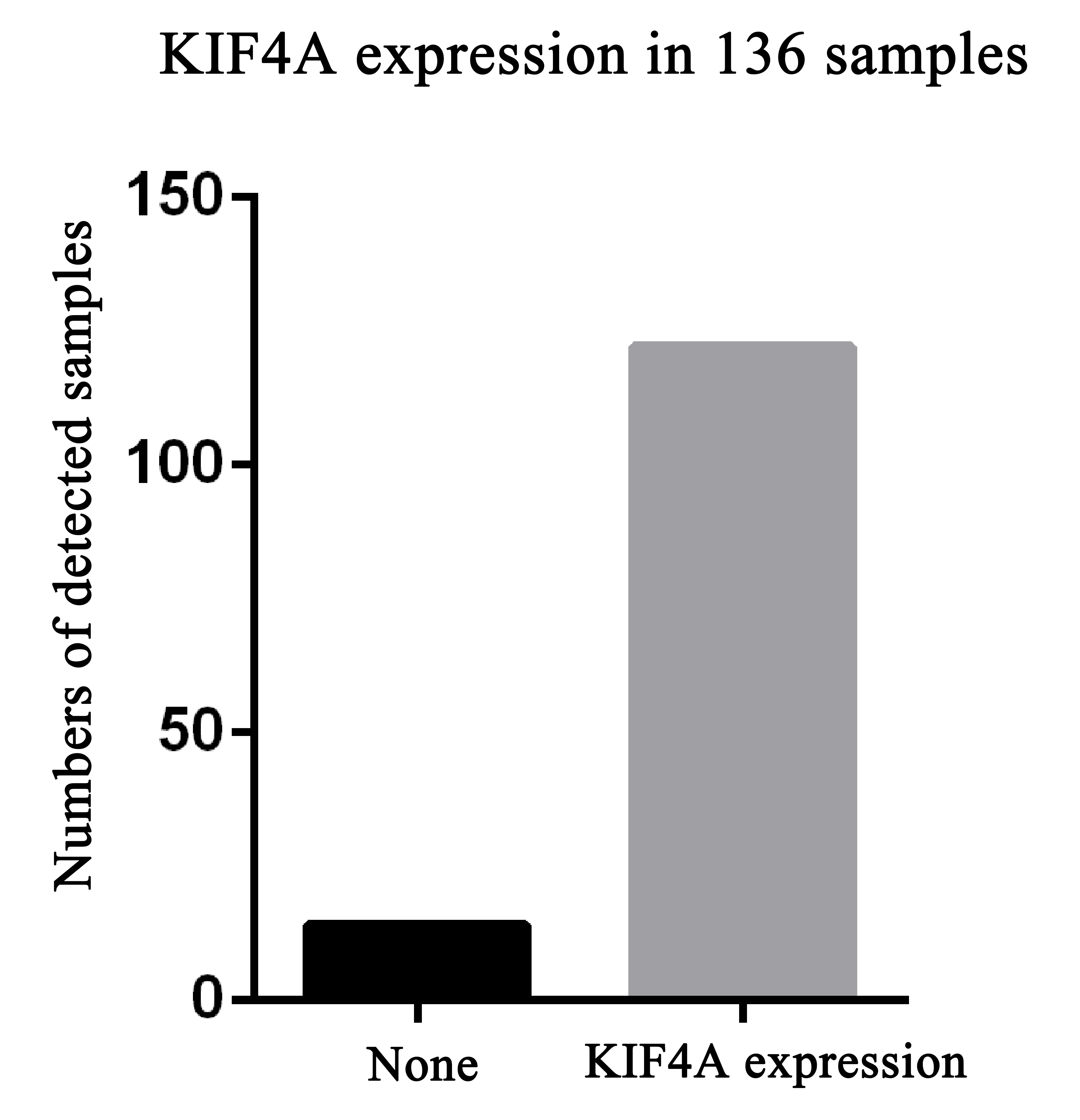

Supplement: Supplementary file 2 — Revised Supplementary Figure S2 [file 41419_2017_114_MOESM2_ESM.tif]

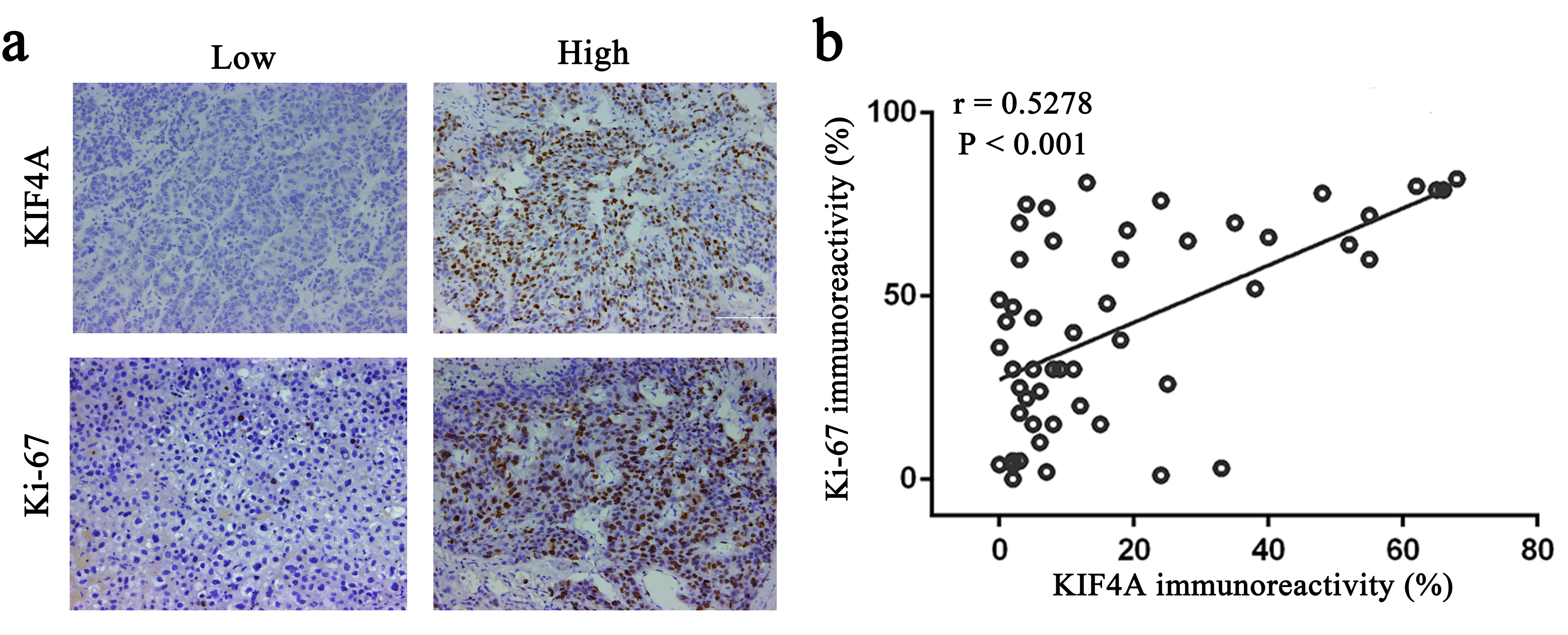

Supplement: Supplementary file 3 — Revised Supplementary Figure S3 [file 41419_2017_114_MOESM3_ESM.tif]
