# Supplementary material for: Upregulation of kinesin family member 4A enhanced cell proliferation via activation of Akt signaling and predicted a poor prognosis in hepatocellular carcinoma
Source: Cell Death Dis. 2018 Feb 2;9(2):141. doi: 10.1038/s41419-017-0114-4 (PMC5833581; doi:10.1038/s41419-017-0114-4)
Supplement: Supplementary file 4 — Supplementary Figure Legends [file 41419_2017_114_MOESM4_ESM.docx]

**Supplementary Figure Legends**

**Supplementary Figure S1**. Representative image showed that KIF4A mRNA was overexpressed in HCC tissues compared with adjacent non-neoplastic controls in four selected datasets from Oncomine database. The differences between the normal and tumor groups were compared using t-test.

**Supplementary Figure S2**. Summary of KIF4A expression in 136 HCC tissues.

**Supplementary Figure S3**. Expression levels of KIF4A and Ki-67 showed positive correlation in HCC tissue. (a) Immunohistochemical staining of KIF4A and Ki-67 protein expressions in 53 HCC tissues. Representative images were shown. Scale bar: 100 μm. (b) Scatterplot of immunoreactivity scores of Ki-67 versus KIF4A with regression line showed a positive correlation.
